# Health-related vulnerability to climate extremes in homoclimatic zones of Amazonia and Northeast region of Brazil

**DOI:** 10.1371/journal.pone.0259780

**Published:** 2021-11-11

**Authors:** Lara de Melo Barbosa Andrade, Gilvan Ramalho Guedes, Kenya Valeria Micaela de Souza Noronha, Cláudio Moisés Santos e Silva, Jéferson Pereira Andrade, Albert Smith Feitosa Suassuna Martins

**Affiliations:** 1 Department of Atmospheric and Climate Sciences, Universidade Federal do Rio Grande do Norte (UFRN), Natal, Rio Grande do Norte, Brazil; 2 Departament of Demography, Center for Development and Regional Planning, Universidade Federal de Minas Gerais, Belo Horizonte, Minas Gerais, Brasil; 3 Departament of Economics, Center for Development and Regional Planning, Universidade Federal de Minas Gerais, Belo Horizonte, Minas Gerais, Brasil; 4 Departament de Statistics, Universidade Federal de Minas Gerais, Belo Horizonte, Minas Gerais, Brasil; National Sun Yat-sen University, TAIWAN

## Abstract

Amazonia and the Northeast region of Brazil exhibit the highest levels of climate vulnerability in the country. While Amazonia is characterized by an extremely hot and humid climate and hosts the world largest rainforest, the Northeast is home to sharp climatic contrasts, ranging from rainy areas along the coast to semiarid regions that are often affected by droughts. Both regions are subject to extremely high temperatures and are susceptible to many tropical diseases. This study develops a multidimensional Extreme Climate Vulnerability Index (ECVI) for Brazilian Amazonia and the Northeast region based on the Alkire-Foster method. Vulnerability is defined by three components, encompassing exposure (proxied by seven climate extreme indicators), susceptibility (proxied by sociodemographic indicators), and adaptive capacity (proxied by sanitation conditions, urbanization rate, and healthcare provision). In addition to the estimated vulnerability levels and intensity, we break down the ECVI by indicators, dimensions, and regions, in order to explore how the incidence levels of climate-sensitive infectious and parasitic diseases correlate with regional vulnerability. We use the Grade of Membership method to reclassify the mesoregions into homoclimatic zones based on extreme climatic events, so climate and population/health data can be analyzed at comparable resolutions. We find two homoclimatic zones: Extreme Rain (ER) and Extreme Drought and High Temperature (ED-HT). Vulnerability is higher in the ED-HT areas than in the ER. The contribution of each dimension to overall vulnerability levels varies by homoclimatic zone. In the ER zone, adaptive capacity (39%) prevails as the main driver of vulnerability among the three dimensions, in contrast with the approximately even dimensional contribution in the ED-HT. When we compare areas by disease incidence levels, exposure emerges as the most influential dimension. Our results suggest that climate can exacerbate existing infrastructure deficiencies and socioeconomic conditions that are correlated with tropical disease incidence in impoverished areas.

## Introduction

Population vulnerability is a multidimensional concept that depends on the context in which individuals are exposed to adverse conditions, such as climatic, environmental, sociodemographic, and health factors [1–3]. The severity of vulnerability experienced depends on the interplay among extreme climatic events, sociodemographic susceptibility, and a society’s adaptive capacity [2, 4]. In the short and medium run, population vulnerability can be mitigated by public policies, such as poverty alleviation, household infrastructure improvements, and access to adequate healthcare services. On the other hand, climate exposure requires long-term interventions that involve multiple strategies and different stakeholders [5–7]. Since climate does not respect political boundaries, any steps taken towards environment sustainability require political agreements on a global scale, as well as some level of domestic economic sacrifice among select demographic and economic sectors [8, 9]. Because of the complex coordination required, structural changes in economic production and human occupation that reduce climatic exposure are considered the most difficult targets on the vulnerability reduction agenda [2, 4, 10].

Extreme weather events are the most common measurements of climate exposure and their effects are diverse and usually more prominent in highly vulnerable families and societies [11, 12]. They can leave families permanently or temporarily without access to basic infrastructure and, in some cases, can displace entire communities. The damages can even affect subsistence activities, such as family farming and fishing, which reinforce the poverty trap. At the societal level, extreme weather events can destroy public infrastructure and, ultimately, affect the economy [13, 14].

Extreme weather events can directly affect human health by causing death, physical injury, illness, or mental health problems. For instance, in 2011 alone, the excessive rainfall and the resulting landslides in Rio de Janeiro affected around 300.000 individuals and resulted in more than 900 deaths [15]. However, the severity of the health impacts due to extreme climate events depends on how well-equipped each society is in terms of infrastructure [16]. High-income areas frequently impacted by extreme weather events, such as New Zealand and Japan (hurricanes and earthquakes) and the Netherlands (floods), have shown little loss of human life when compared to hard-hit low and middle-income areas, such as Thailand and the Philippines [17–19]. Even in developed countries, such as the United States, impoverished areas can be severely affected by extreme weather events [20, 21].

Extreme climate events can also have direct health effects, such as physical injuries and mental health impacts [22]. Several meteorological conditions including variations in temperature, relative humidity, wind speed, and air pressure can also cause health problems [22, 23]. Increasing average daily temperatures or changes in precipitation patterns or intensity can affect pathogen and disease vector development. Sustained temperature increases can shorten parasites’ life cycles, such as of plasmodium, while changes in precipitation volume can contribute to disease vectors’ spread [16, 24, 25]. As shown by Lapouble et al. [26], flooding can affect the movement of pathogens from environmental reservoirs to both surface and groundwater, contaminating hydric systems. High temperatures and heavy rains contribute to an increase in pathogens’ reproduction rates and survival, while long periods of drought favor their accumulation through fecal deposition [16].

Climate interactions with ecosystems, water, and biodiversity, as well as changes in land use, can lead to environmental degradation, affecting both food and water availability and quality [27]. Water shortages can exacerbate the incidence of infectious diseases due to worsened sanitary conditions and drinking water access. Empirical evidence has shown that long periods of drought have increased the number of dengue cases due to inadequate water storage [1, 28]. There is also growing evidence concerning the health consequences of migration induced by droughts, both in Brazil and elsewhere [29, 30]. Migrants from endemic areas may carry communicable diseases as they move to new areas and could also be infected when moving to endemic areas, which could result in an upsurge in migration-induced morbidity and mortality [29].

**Fig 1:**
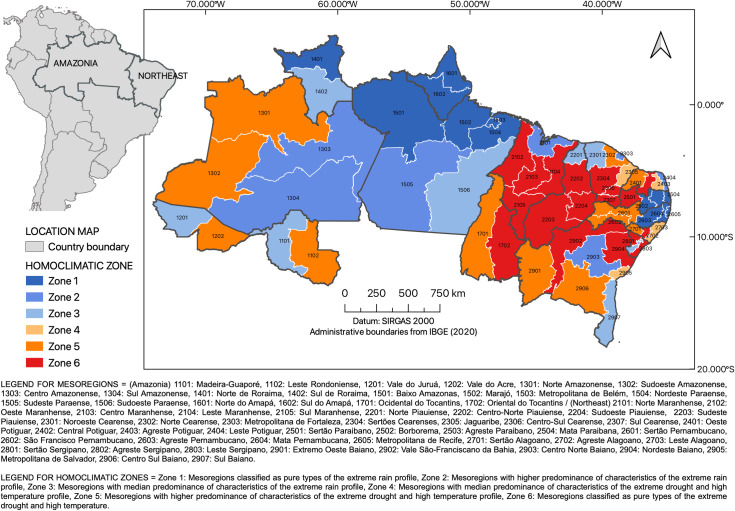
Spatial distribution of homoclimatic zones across the mesoregions in the Brazilian Amazon and in the Northeast.

Recently, droughts, floods, cold snaps, and heat waves have been recorded in different Brazilian regions, such as the drought that affected the South of Brazil in 2008 [13] and the water crisis due to a precipitation deficit in São Paulo in 2014 and 2015 [31]. In particular, Amazonia (AMZ) and the Northeast of Brazil (NEB), represented in the map in Fig 1, have experienced the most intense and frequent extreme weather events in the country, encompassing both periods of torrential rainfall and severe droughts [13, 32–35]. These extreme events are often responsible for endemic diseases, intensifying the vulnerability of local populations [2, 4]. In addition, these regions possess the highest climate-sensitive infectious disease rates in the country [36].

The Brazilian AMZ is characterized by a hot and humid climate. However, as the climate conditions are modulated by ocean-atmosphere mechanisms, the region experiences total rainfall above or below climatological averages that causes extremely humid or dry days [37, 38]. In recent years, the region has experienced alternating periods of heavy rainfall (2009, 2011, 2012, and 2014) and severe droughts (2005 and 2010) that affected the living conditions of thousands of families and increased the incidence of climate-sensitive diseases [39–43].

In turn, the NEB possesses the lowest water availability among the Brazilian regions, particularly in semiarid areas [44]. It experiences noticeable interannual precipitation variations, alternating between extremely dry and extremely rainy years [45]. Droughts form a part of the region’s natural climatic variability and recurrently affect the population, especially the most vulnerable inhabitants living in semiarid areas. Constant water shortages affect the agricultural sector, increasing food insecurity risks and worsening socioeconomic conditions, as the region is highly dependent on family farming [44]. Between 2012 and 2016, the NEB experienced its worst drought in the last 50 years, which affected 83% of the cities [46, 47]. Important economic sectors, such as agriculture and livestock, have also suffered significant losses [46]. The depletion of important water sources has triggered a number of hazardous conditions, including water pollution, which has contributed to the increased incidence of infectious and parasitic diseases [48]. On the other hand, many major Northeastern cities, especially those located in coastal areas, have suffered from heavy rainfall episodes. These events, in conjunction with poor infrastructure and unplanned urbanization, have resulted in severe flooding and adverse health consequences for the general population [49].

The aim of this paper is twofold. First, we define homoclimatic areas that address the longstanding difficulty in the population-climate literature of adequately matching climate data resolution with the administrative-political boundaries used in population data. Second, we provide Alkire-Foster-based [50] estimates of health vulnerabilities to climate extremes on a microregion level for the homoclimatic zones in the Brazilian AMZ and the NEB. These are the two most climate-sensitive regions and are home to the poorest populations in the country. Our Extreme Climate Vulnerability Index (ECVI) encompasses the three most commonly acknowledged dimensions in climate vulnerability literature–exposure, susceptibility, and adaptive capacity [4]. In order to understand how climate vulnerability relates to population health, we estimated the ECVI for different infectious disease incidence levels and broke down the index by the contribution of each of its three dimensions.

Earlier research has already estimated multidimensional indices of health vulnerability to climatic conditions in Brazil at both the national [1] and local levels, encompassing studies focused on the Northeast [51], semiarid areas [30], and specific Brazilian states, including Minas Gerais [10], Amazonas [2], and Rio de Janeiro [52]. The proposed ECVI builds upon these prior efforts in multiple ways. To the best of our knowledge, this is the first study to estimate a vulnerability index for the two most important Brazilian regions in terms of climatic regulation and ecosystem diversity. It is also the first study to explicitly incorporate vulnerability levels and intensity using the type of climatic data that most adequately captures climate-related health vulnerabilities. Furthermore, our analysis is based upon climate data that uses the homogenous climate zones proposed by Oliveira et al [45], Silveira Marinho et al [53], and Santos et al [37, 38]. In addition, instead of considering classical geographic classifications, we used the GoM method to reclassify Brazilian mesoregions into homoclimatic zones based on extreme climatic events. This reclassification is important as climate does not adhere to political-administrative boundaries. Several studies have previously attempted to define homogeneous climate regions based on different indicators and multivariate techniques [53]. For Brazil, some studies have proposed homoclimatic regions for the Northeast [45, 54, 55], for the South and the Southeast [56], and for the Amazon [37]. Silveira Marinho et al [53] is the sole study to estimate homogeneous regions for the entire country. However, their homogeneous subregions are based on meteorological variables, which are not the most suitable type of data to analyze climate-related health vulnerabilities.

## Materials and methods

### Data

In this study we examine two particular Brazilian regions–the NEB and AMZ, combining epidemiological, demographic, socioeconomic, and climate data. Meteorological data are part of a joint project between the University of Texas (USA) and the Universidade Federal do Espírito Santo (Brazil) and are available at https://utexas.box.com/Xavier-etal-IJOC-DATA. The database contains a rich set of meteorological variables, such as precipitation, wind, minimum and maximum temperatures, relative humidity, and evapotranspiration. These variables are organized in a regular 0.25° x 0.25° grid and cover the entirety of Brazil. Detailed procedures concerning meteorological data extraction and manipulation are described in Xavier et al. [57].

We selected one grid point for the precipitation and temperature variables from each homogeneous precipitation region for all 62 mesoregions (20 in AMZ and 42 in the NEB), as defined by the political-administrative divisions established by the Brazilian Institute of Geography and Statistics (IBGE) [58, 59].

Population health conditions were proxied using climate-sensitive infectious and parasitic disease rates (Chapter 1 of the International Classification of Diseases, 10th edition), including diseases that bear direct and indirect relation with climate conditions [60]. This health proxy is derived from the administrative health records provided by the Brazilian Hospital Information System (SIH in Portuguese), which includes all inpatient care supplied by the Brazilian Public Health Care System (SUS in Portuguese). SUS hospitalizations represent 66% of all tertiary care in the country, and the data is publicly available, which makes it the most used information source for conducting health-related analyses [61, 62]. Health data were recorded at the mesoregion level according to patients’ places of residence, rather than the location of inpatient admission.

We measured disease counts as a 5-year average (2008–2012) based upon the 2010 population because the probability of these events’ occurrence is relatively low, even at the mesoregion level. This strategy contributes to alleviating the distortions usually present when rates are calculated for small areas or when there are seasonal health variations. Mesoregions were then classified into quintiles based on the 2010-centered hospitalization rates. The first quintile defines low incidence areas, whereas the fourth and fifth quintiles represent areas with high levels of disease incidence. We used population size from the 2010 IBGE Demographic Census to calculate climate-sensitive disease rates and the proxies for demographic, socioeconomic, and infrastructure conditions.

All population data, including administrative health records and Census data, are publicly available and none of them contains information which allows identification of respondents or patients.

### Definition of homoclimatic zones

The use of mesoregions as a unit of analysis can generate a loss of climatic spatial variability, especially in the very large and diverse Amazonian region. However, previous studies have shown consistently homogeneous levels of annual rainfall distribution throughout the Brazilian AMZ [37] and the NEB [45]. Although the number of mesoregions is relatively higher than the climatic variability in both areas, homogeneous rainfall regions and mesoregions do not necessarily spatially coincide. Furthermore, climate regimes are not factored into the organizational logic of socioeconomic, demographic and health data, which respect traditional political and administrative boundaries.

To correct the political/climatic boundary mismatch and refine our climate/social spatial resolution we use the Grade of Membership (GoM) method. GoM is a fuzzy cluster method that allows observations in a multidimensional dataset to have multiple memberships, thus modeling the degree of unobserved heterogeneity at the mesoregion level [63]. There are two parameters to be estimated. The first parameter, *λ*_*kjl*_, is the probability that a mesoregion belonging to extreme profile ‘*k*’ will have a particular response in the ‘*l*’-th category of a ‘*j*-th indicator, given the known score *g*_*ik*_. For instance, if *λ*_121_ = 0.52, then a mesoregion that completely belongs to *k* = 1 has a probability of 0.52 to have a particular response in *l* = 1 of variable *j* = 2. The *g*_*ik*_ score is the part of the classification technique that defines it as a fuzzy method as it estimates ‘*k*’ degrees of pertinence profiles (cluster) for each mesoregion. If *g*_10,1_ = 0.8, for example, then the mesoregion *i* = 10 possess an 80% membership (proximity) to *k* = 1. Model identification is subject to two restrictions:

$$g_{ik}\geq 0\;for\;each\;i$$ (1.1)

$$\sum_{k|i}g_{ik}=1$$ (1.2)

For the same profile, *k*, and the same variable, *j*, the parameter *λ*_*kjl*_ is normalized such that ∑_*l*|*jk*_*λ*_*kjl*_ = 1. The likelihood structure is based on the mesoregion-level conditional probability for a particular response of the ‘*i*’-th mesoregion to the ‘*l*’-th category of the ‘*j*’-the variable, represented by *P*(*Y*_*ijl*_ = 1) = ∑_*k*_*g*_*ik*_*λ*_*kjl*_. The probability model, based on a random sample, corresponds to *E*(*Y*_*ijl*_), with *g*_*ik*_ a strictly positive known parameter, by assumption. This probabilistic structure on a random sample results in a likelihood function with the following multinomial form [64]:

$$L(Y_{ijl})=\int \prod_{i=1}^{I}\prod_{j=1}^{J}\prod_{l=1}^{L}{\left(\sum_{k=1}^{K}g_{ik}\lambda_{kjl}\right)}^{y_{ijl}}\;f(g_{i1},\ldots ,g_{iK}|\alpha )dg_{i1}\ldots dg_{iK}$$ (2)

This structure maximizes the likelihood in *λ*_*kjl*_ and ***α***. This formulation assumes that *g*_*ik*_ are random variables, i.i.d. with a joint density function *f*(*g*_*i*1_,…,*g*_*iK*_|*α*). So, different from Manton et al. [63], we do not estimate *g*_*ik*_ directly from the data, but indirectly by integrating out *f*(.|*α*) to get the unconditional likelihood. Since 0≤*g*_*iK*_≤1 and given restriction 1.2 above, a natural choice for *f*(.|***α***) is the Dirichlet distribution. The ***α*** vector is a set of parameters governing the Dirichlet distribution. In our formulation, we consider a reparameterization of *α* = (*α*_1_,…,*α*_*K*_) with $\alpha_{0}=\sum_{k=1}^{K}\alpha_{k}$ and *ξ* = (*ξ*_1_,…,*ξ*_*K*_), where *ξ*_*k*_ = *α*_*k*_/*α*_0_, as described in Erosheva et al. [65]. In this parametrization strategy the components of vector *ξ* can be interpreted directly considering the proportion of item responses that belong to each extreme profile, while *α*_0_ represents the spread of the membership distribution *g*_*iK*_. As a result, a lower *α*_0_ indicates a better estimate of extreme profiles.

To avoid the integration in Eq (2), we use the Bayesian procedure of Markov Chain Monte Carlo (MCMC) proposed in Erosheva et al. [65]. The posterior distributions of model parameters were obtained via the Gibbs sampler based on 5,000 iterations after a 2,500 burn-in period. The Bayesian formulation of GoM model used in this study was performed using Stata 14.0 based on the *ugom* command [66].

In our GoM model we use five extreme temperature indices and two extreme precipitation indices produced by the Climdex Project [59, 57]. All indicators, which correspond to J variables from our GoM equations, are defined using the data that cover the period from 1980 to 2013. The extreme temperature indices include TXx (monthly maximum value of daily maximum temperature–C^o^), TNx (monthly maximum value of daily minimum temperature–C^o^), TX90p (percentage of days when TX > 90th percentile–warm days), TN90p (percentage of days when TN > 90th percentile–warm nights), and DTR (daily temperature range equivalent to the monthly mean difference between TX and TN–C^o^). Extreme precipitation indices include CDD (maximum number of consecutive days when rainfall <1 mm–dry spells) and R99p (annual total daily precipitation that exceeded the 99th percentile–extremely wet days in mm). All extreme climate indexes were treated as quintiles. Each quartile corresponds to the *l*-th category of the *j*-th index from our GoM equations.

### Extreme climate vulnerability index (ECVI)

We applied the Alkire-Foster (AF) method [50] to select mesoregion indicators, in order to create a multidimensional index of vulnerability to climate extremes, named the Extreme Climate Vulnerability Index (ECVI). This index was estimated for the overall study area and for the homoclimatic zones defined by GoM, weighted by their respective population sizes. We further decomposed the ECVI by different levels of infectious disease incidence, in order to understand how health correlates with climatic and socioeconomic indicators in our study areas.

Formally, the ECVI is defined by the interaction between the censored (multidimensional) deprivation headcount (CH) and the censored deprivation intensity (DI). The CH represents the proportion of mesoregions that are simultaneously deprived of at least *k* among a total of *I* indicators. In turn, the DI corresponds to the average proportion of indicators that mesoregions are deprived of among mesoregions deprived of at least *k* indicators.

Indicators can be used directly or grouped by dimension. In this study we used exposure, susceptibility, and adaptive capacity as the three dimensions that define the ECVI. The indicators that comprise each dimension are shown in Table 1. Exposure is proxied by the five extreme temperature indices and two extreme precipitation indices used in the GoM method, with a mesoregion classified as deprived if each of its indicators has a value higher than the lower bound of its fourth quartile. The susceptibility dimension is measured by five indicators, which encompass mesoregions where the elderly proportion of the population surpasses the lower bound of the fourth quartile, mesoregions where children comprise a proportion of the population above the lower bound of the fourth quartile, mesoregions with an average monthly per capita income below R$255.00 (½ of the 2010 minimum wage), mesoregions where the proportion of poor individuals is above the lower bound of the fourth quartile, and mesoregions where the population proportion of literate adults is below the first quartile. The adaptive capacity dimension was proxied by six indicators, with a mesoregion being considered deprived if its values for each of the indicators are lower than its first quartile. The six indicators used are the proportion of households with adequate sewage (either sanitary sewer or septic tank), the proportion of households with an adequate water supply, the proportion of households with garbage collection, urbanization rate, the proportion of individuals covered by the Family Health Strategy (primary care coverage), and the rate of hospitalization beds per 100,000 inhabitants. All individual indicator cutoffs are described in S1 Fig and S1 Table.

**Table 1:** Cut-offs and weights attributed to each indicator of ECVI.

Dimension/Indicator	Deprivation cut-off	Weight
**Exposure**		
Monthly maximum value of daily maximum temperature (°C)	4th quartile	0.0476
Monthly maximum value of daily minimum temperature (°C)	4th quartile	0.0476
Percentage of warm days	4th quartile	0.0476
Percentage of warm nights	4th quartile	0.0476
Daily temperature range	4th quartile	0.0476
Dry spell	4th quartile	0.0476
Extremely wet days	4th quartile	0.0476
*Total exposure dimensional weight*		0.3333
**Susceptibility**		
Higher proportion of elderly (over 60 years old)	4th quartile	0.0667
Higher proportion of children (less than 5 years old)	4th quartile	0.0667
Proportion of households with low income	Average per capita income < R$296.8 (US$ 214.1 ^(1)^)	0.0667
Higher proportion of Poor individuals (household income per capita < ½ minimum wage)	4^th^ quartile	0.0667
Lower proportion of literate adults	1st quartile	0.0667
*Total susceptibility dimensional weight*		0.3333
**Adaptive Capacity**		
Lower proportion of households with adequate sewage	1st quartile	0.0556
Lower proportion of households with adequate water supply	1st quartile	0.0556
Lower proportion of households with garbage collection	1st quartile	0.0556
Lower levels of urbanization	1st quartile	0.0556
Lower primary care coverage (%) (number of individuals registered by the Family Health Strategy)	1^st^ quartile	0.0556
Lower proportion of hospital beds per 100,000 inhabitants	1^st^ quartile	0.0556
*Total adaptive capacity dimensional weight*		0.3333

ECVI: Extreme Climate Vulnerability Index.

(1) Brazilian Currency was converted to the 2010 US dollars exchange rate using the CCEMG—EPPI-Center Cost Converter website (<http://eppi.ioe.ac.uk/costconversion/default.aspx>).

One of the most appealing features of the AF methodology is its ability to incorporate weights to dimensions/indicators that are meaningful to the theory or to public policy interventions and monitoring. In this study we assigned equal weights to the dimensions (1/3 each), but, since the dimensions have different numbers of indicators, we allowed weights to vary by indicator within each dimension without altering the overall dimensional weights. The choice to assign equal weights to the dimensions was taken to capture the sole impact of compositional differences across mesoregions. Furthermore, other studies using different methodologies assign equal weights to dimensions to avoid entangling the varying theoretical dimensional importance with units’ empirical compositional heterogeneity [53]. Thus, the difference among final indicator weights is a pure reflection of data availability for how we measured the dimensions, rather than how we proxied their relevance [10, 50]. This weighting structure is shown in Table 1.

In accordance with Alkire and Foster [50], we established different indicator proportions (*k*_*p*_ = *k*/*I*) to classify mesoregions as deprived or not. A key decision to make when estimating AF-based indices is the choice of the ideal value for *k*_*p*_, as it directly affects the level and intensity of the multidimensional index, as well as the contribution of each dimension/indicator. In this study we use two criteria, encompassing an examination of the regions of the ECVI, CH, and DI curves that are relatively flat and whether the ECVI curve for a particular homoclimatic zone sits above the other overall feasible values of *k*_*p*_. While the first criterion is a type of sensitivity analysis, the second is better known as dominance analysis. Using the first criterion, we define *k*_*p*_ = 25% as the vulnerability cutoff, since it represents the point at which the disturbance in vulnerability trends is locally minimized. The second criterion allows us to show that the level of vulnerability for one homoclimatic region is always higher than for the other, regardless of the choice of *k*_*p*_. We developed an automated local optimization criterion that permits us to choose the minimum variance of the selected index (ECVI, CH, or DI) calculated on the *t* forward points from *t = p*. When more than one group is compared (as in the dominance analysis), we also included an optimization criterion for the *k*_*p*_ selection; however, instead of looking at the optimal *k*_*p*_ for each curve, we minimize the average forward local variance across groups. The optimization procedures described must be used as an additional tool and not as the sole criterion, since they depend on the number of cuts in the *k*_*p*_ domain, the number of groups to be compared, and the final vulnerability level to be addressed. The *k*_*p*_ = 45% suggested by the optimization criterion in this particular application would lead to an excessively small number of mesoregions. The *k*_*p*_ = 25% chosen seems more adequate as it represents the point where the CH and the DI intersects, while still allowing for a reasonable number of regions to be analyzed in the pool of vulnerable regions. The sensitivity and dominance analyses are available in the S2 Fig and S2.1-S2.3 Tables in S2 Table.

As a subsequent step of our sensitivity analysis, we estimated the ECVI and its indicators, in addition to its dimensional and regional decomposition, for a 30% cutoff; however, the results are quite similar to *k*_*p*_ = 25% (S3.1-S3.2 Tables in S3 Table). Although a 35% to 45% *k*_*p*_ interval looks reasonably flat, such high cutoffs would produce a very small number of multidimensionally deprived mesoregions (S2 Fig and S2.1-S2.3 Tables in S2 Table). For instance, using *k*_*p*_ = 45% would result in less than 3% of Extreme Rain (ER) homogenous climatic mesoregions being classified as vulnerable.

After estimating the ECVI, we decomposed it by the homoclimatic zones to understand how much of the overall vulnerability is due to each study area. If the entire study area *y* (of size *n*) is divided into two subgroups, *y*_1_ (of size *n*_1_) and *y*_2_ (of size *n*_2_), then the ECVI can be expressed as a weighted function of each subgroup as follows:

$$ECVI(y)=\frac{n_{1}}{n}\times ECVI(y_{1})+\frac{n_{2}}{n}\times ECVI(y_{2})$$ (3)

The contribution of subgroup *i* to the overall adjusted ECVI is then:

$$\frac{n_{i}}{n}\times \frac{ECVI(y_{i})}{ECVI(y)},\;for\;i=1,2$$ (4)

Decomposing the ECVI by indicators requires writing it as a function of the relative weight attributed to each indicator and the indicator-specific censored headcount (*CH*_*i*_). If the censored headcount of the *i*-th indicator is denoted by *CH*_*i*_, then the adjusted ECVI can be expressed as:

$$ECVI(y)=\sum_{i}\frac{w_{i}}{I}\times {CH}_{i}$$ (5)

where *w*_*i*_ is the weight attached to the *i*-th indicator. Eq (5) can be easily written to express decomposition by dimension. In this case, a second summation needs to be added, along with the relative weight assigned to each dimension. The contribution of the *i*-th indicator to the overall ECVI is

$$\left(\frac{w_{i}}{I}\right)\times \left(\frac{CH_{i}}{ECVI}\right)\;for\;all\;i=1,\ldots ,I$$ (6)

We used the software R, version 4.0.3, for data manipulation (based on the “tidyverse” suite) and to estimate the ECVI (based on the “survey” and “convey” libraries). Maps were created with the QGIS software, version 3.14. All scripts and data used in this study are available at <https://github.com/epopea/ecvi.git>.

## Results

### Homoclimatic regions

Based on the GoM model, we found two extreme climate profiles (*k* = 2). The Boxplots (S3 Fig) characterizes the extreme profiles regarding their climatic features by means of the posterior distributions of *λ*_*kjl*_ (where *j* = 1,2,…,7 and *l* = 1,2,3,4,5). For most indicators, the boxplots are symmetrical across profiles, suggesting an efficient solution for the number of reference groups. The horizontal line represents a threshold for a characteristic at least 20% higher than in the overall study region. Mesoregions belonging to the first extreme profile are characterized predominantly by extremely wet days (4^th^ and 5^th^ quantiles), low levels (1^st^ quantile) of the monthly maximum value of daily maximum temperature, and either low (1^st^ quantile) or high (5^th^ quantile) percentage of warm days and nights. The second extreme profile, in contrast, comprises mesoregions predominantly characterized by long dry spells (3^rd^, 4^th^ and 5^th^ quantiles), high levels (4^th^ and 5^th^ quantile) of monthly maximum value of daily maximum temperature and of daily temperature range, and median levels (3^rd^ quantile) of monthly maximum value of daily minimum temperature, and percentage of warm days and warm nights.

Based on their characteristics, the first and the second extreme profiles were named Extreme Rain (ER) and Extreme Drought and High Temperature (ED-HT), respectively. Mesoregions were classified into the *k*-th extreme profile if their degree of membership (*g*_*ik*_) were equal or higher than 0.90. This criterion identified 50% of the mesoregions. The remaining 50% partially belonging to more than one extreme profile were classified as mixed profiles based on two additional *g*_*ik*_ cut-off points: 0.75≤*g*_*ik*_<0.90 and 0.50≤*g*_*ik*_<0.75, higher and median predominance of the *k*-th extreme profile characteristics, respectively.

The classification of mesoregions into each extreme zone (ER and ED-HT) is consistent with previous empirical studies on rainfall in the tropical region of South America, which include the Brazilian AMZ and NEB, even using different climatological data [67, 68]. All 18 mesoregions of the extreme ED-HT profile (*g*_*ik*_ ≥ 90%) are located in the semiarid zone of NEB or in the transition areas between AMZ and NEB. The climatic indexes in the semiarid zone are associated with extremes temperatures (TXx) and precipitation deficit (CDD), despite historical registers of sporadic intense rain [45] (Fig 1). The zone also exhibits high DTR due to the low levels of air humidity, a measure of water vapor in the atmosphere, which results in low absorption of longwave radiation emitted by the earth’s surface.

The twelve mesoregions with high predominance of the ED-HT extreme profile are located both in the NEB (7) and in the AMZ (5). Although the mesoregions of the Southwest of AMZ (North Amazonense, Southwest Amazonense and Vale do Acre) have the highest accumulation of annual precipitation (averaging over 3,000 mm), its distribution is uniform throughout the year, with few outliers [38]. The East Rondoniense and Western Tocantins mesoregions, in turn, are part of the border between the Amazon biome and the Brazilian Cerrado, an area known as the Arc of Deforestation. These areas have been severely impacted by non-sustainable land use systems [69, 70], with serious hydroclimatic consequences [71]. The four remaining mesoregions with median predominance of the ED-HT extreme profile are all located in the NEB.

The extreme profile ER (*g*_*ik*_ ≥ 90%) comprises 13 mesoregions from which seven are located in the Brazilian AMZ, mainly in the north, and six are part of the coast from the NEB. In the north of Brazilian AMZ, extreme precipitation events are associated with the occurrence of Mesoscale Convective Systems which have a Squall Line configuration [72]. The six NEB mesoregions from the coast area are located further east of the region, between Mata Paraibana and Agreste Pernambucano. They are impacted by extreme precipitation events that stem from east wave disturbances and the interaction between the runoff and the topography in the Borborema Plateau [45, 73].

Among the mesoregions with high predominance of the extreme ER profile characteristics, three are located in the central region of the AMZ (Centro Amazonense, Sul Amazonense and Sudeste Paraense) and four are in the NEB, of which three are coastal areas (Norte Maranhense, Metropolitana de Fortaleza and Leste Potiguar) and one is located in the central region of Bahia (Centro-Norte Baiano). Mesoregions with medium predominance of the extreme ER profile features are equally distributed between NEB and the Amazon, four in each of them. The majority of the NEB mesoregions classified into the high and medium mixed ER profiles are located on the coast. Mesoregions located in the North of the NEB are affected by the meridional migration from the Intertropical Convergence Zone [74]. The Southern Bahia mesoregion, in turn, is influenced by the frontal systems from mid-latitudes or events in the South Atlantic Convergence Zone that operate in the region during the summer [75, 76].

For the AF index decomposition purposes, we reclassified the mixed (high and medium) profiles into their respective extreme profiles (ER and ED-HT). Neither the ED-HT nor ER zones are more clearly affected by exposure to adverse climatic conditions. For example, among the ED-HT mesoregions precipitation levels are significantly lower than in their ER counterparts. Consequently, the ED-HT mesoregions experience an additional 39.5 consecutive days with less than 1 mm of rainfall. Conversely, ER areas has 93.9 more extremely wet days than the ED-HT zone, on average (S1 Fig and S1 Table).

On average, ER and ED-HT regions share similar sociodemographic characteristics (S1 Fig and S1 Table). Based on the selected indicators from S1 Table, it is not possible to clearly identify the most vulnerable homogeneous climatic zone. Statistically significant differences are only observed for the proportion of elderly, the proportion of households with garbage collection, urbanization rate, and primary health coverage. The ED-HT mesoregions are more vulnerable with regards to garbage collection coverage and urbanization rate. In contrast, primary health coverage is lower among the ER mesoregions (S1 Fig and S1 Table).

The ED-HT mesoregions present a slighter older population, which reflects the composition of this homogeneous climatic area that comprises the majority of Northeast mesoregions. Even though older populations are associated with higher levels of socioeconomic development, elderly individuals tend to be more vulnerable to climate-sensitive diseases [77]. In addition, the aging population process experienced by the Brazilian Northeast has more to do with the age selectivity of out and return migration, rather than with improvements in local socioeconomic conditions [78].

Hospitalization rates due to infectious and parasitic diseases are approximately 815 and 708 per 100,000 inhabitants for the ED-HT and ER homogeneous climatic regions, respectively, although this difference is not statistically significant (S1 Fig and S1 Table). Infectious and parasitic disease incidence rates vary from 1,705 per 100,000 inhabitants in the Southeast Piauiense mesoregion, in sharp contrast with a rate of 199 per 100,000 inhabitants in the Sertão Sergipano mesoregion, both classified in the ED-HT group.

### Extreme climate vulnerability index (ECVI)

Average regional differences sometimes overestimate vulnerability when dimensions are not simultaneously considered. This can occur if a region is considered deprived for one or two indicators, but does not show enough simultaneous deprivation to reach multidimensional vulnerability. Table 2 shows our ECVI, censored deprivation headcount (CD), and deprivation intensity (DI) estimates for the overall study area and for each region. According to our results, 11% of all 62 mesoregions are classified as deprived for at least 25% of the indicators weighted by their intensity. Among the mesoregions classified as ED-HT, the ECVI is 12%, a figure 31% higher than the estimated multidimensional vulnerability for the ER homoclimatic zone. In addition, the contribution of the ED-HT area to the overall ECVI is about 53% (Table 2). The ED-HT climatic zone comprises almost 55% of the mesoregions, but accounts for only 46% of the population in the overall study area. If the population of the ED-HT zone were larger, its contribution would easily be even higher.

**Table 2:** ECVI, Censored Headcount and Vulnerability Intensity for the overall and each homoclimatic region (k = 0.25).

Indicator	Overall		ER		ED-HT	
	Index	SE	Index	SE	Index	SE
ECVI	0.111	0.024	0.097	0.033	0.127	0.036
Censored Headcount	0.309	0.070	0.284	0.106	0.337	0.093
Vulnerability Intensity	0.359	0.022	0.342	0.033	0.376	0.023
Contribution of each region to the Overall ECVI (%)			46.9	0.118	53.1	0.118

ER: Extreme rain zones in the Brazilian Amazon and Northeast region; ED-HT: Extreme drought and high temperature in the Brazilian Amazon and Northeast region; ECVI: Extreme Climate Vulnerability Index; SE: Standard Error.

According to the CD, 34% of the ED-HT mesoregions are considered deprived for at least 25% of indicators, while, in the ER areas, this value is 28%. Besides its relatively more deprived mesoregions, DI is also higher in the ED-HT zone, with an average of 38% of indicators indicating deprivation intensity, compared with 34% in the ER zone (Table 2).

**Fig 2:**
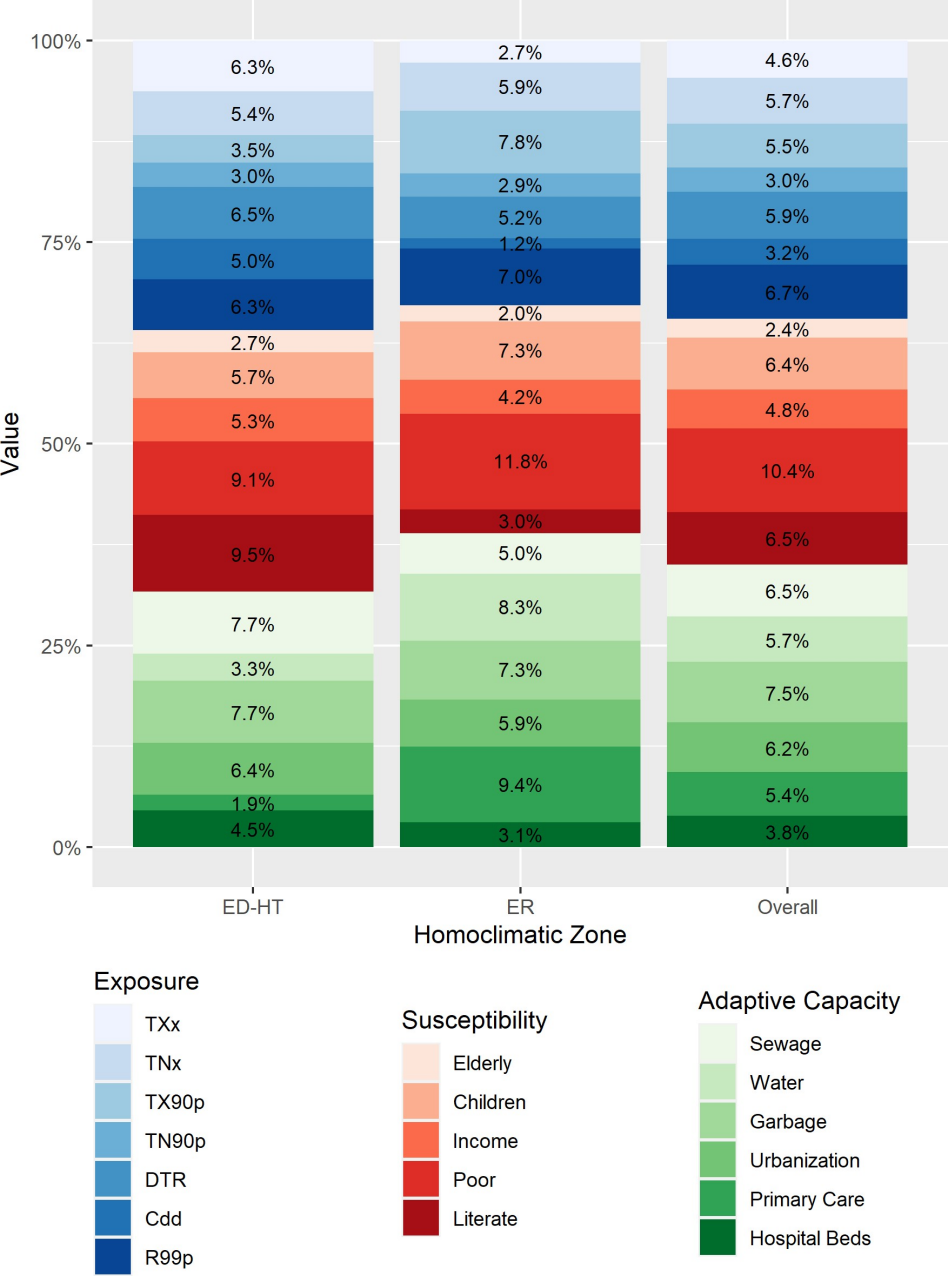
Percent contribution of each indicator to the ECVI by dimension and homoclimatic region (k = 0.25). TXx: Monthly maximum value of daily maximum temperature (oC), TNx: Monthly maximum value of daily minimum temperature (oC); TX90p: Percentage of warm days; TN90p: Percentage of warm nights; DTR: Daily temperature range; Cdd: Dry spell; R99p: Extremely wet days; ER: Extreme rain zones in the Brazilian Amazon and Northeast region; ED-HT: Extreme drought and high temperature in the Brazilian Amazon and Northeast region; ECVI: Extreme Climate Vulnerability Index.

The ECVI’s dimensional decomposition shows that adaptive capacity (35%) and exposure (35%) are the two most important contributors to the vulnerability level in the combined regions (Fig 2). Together, access to garbage collection, adequate sewage and adequate water supply explain 20% of the ECVI. Accordingly, public policies that scale up access to these services to 100% of households would reduce multidimensional vulnerability by 20%, from 11% to 9%.

Regional decomposition reveals interesting patterns (Fig 2). While, in the ED-HT areas, the three dimensions more or less equally contribute to explaining vulnerability, in the ER zone, the contribution of adaptive capacity proves to be the most prominent (39%). Among the ED-HT mesoregions, 36% of vulnerability is explained by exposure to adverse climatic conditions. Low precipitation levels (dry spells), high temperatures, and high daily temperature range stand out as the main indicators explaining vulnerability in the ED-HT relatively to the ER zone. Susceptibility explains a slightly higher share of the ED-HT’s ECVI (32%), with the low proportion of illiterate individuals alone explaining 10%. Even though the proportion of elderly individuals explains very little the multidimensional vulnerability, its contribution for the ED-HT’s ECVI (3%) is higher than among the ER mesoregions (2%). The regional contribution of adaptive capacity to the ED-HT’s ECVI is around 32%, particularly due to low garbage collection coverage and barriers to adequate sewage access. Each of these indicators explains alone around 8% of multidimensional vulnerability. The rate of hospitalization beds plays an important role to the ED-HT multidimensional vulnerability, explaining 5% against 3% among the ER mesoregions.

The large contribution of adaptive capacity (39%) to the ECVI in the ER homoclimatic zone reflects precarious access to adequate water and the low proportion of primary care coverage. If access to both services increased to 100% of the households the ECVI would decrease 18%, from 10% to 8%. Exposure to adverse extreme climatic events is the second most important component (33%). Among the exposure indicators, relatively high proportion of extremely wet day (7%) and high percentage of warm days (8%) are the most important indicators explaining vulnerability in the region.

Table 3 shows multidimensional estimates according to the incidence of climate-sensitive infectious and parasitic diseases. Vulnerability is greater in mesoregions with high disease incidence (14%), compared with those displaying low disease incidence (4%). Regional disparities in multidimensional vulnerability disappear when the analysis is broken down by level of disease incidence. Among areas of high disease incidence, the ECVI is as large as 14% and 13% in the ED-HT and ER homoclimatic zones, respectively. For low disease incidence, the ECVI is around 4% in both zones.

**Table 3:** ECVI, Censored Headcount and Vulnerability Intensity for the overall and homoclimatic regions by level of climate-sensitive diseases (k = 0.25).

Indicator	Low incidence		High incidence	
	Index	SE	Index	SE
	Overall			
ECVI	0.044	0.026	0.136	0.043
Censored Headcount	0.115	0.070	0.348	0.106
Vulnerability Intensity	0.378	0.040	0.392	0.024
	**ER**			
ECVI	0.045	0.039	0.132	0.065
Censored Headcount	0.134	0.116	0.335	0.163
Vulnerability Intensity	0.336	0.005	0.395	0.031
	**ED-HT**			
ECVI	0.042	0.039	0.140	0.058
Censored Headcount	0.094	0.085	0.361	0.142
Vulnerability Intensity	0.444	0.076	0.389	0.037

ER: Extreme rain zones in the Brazilian Amazon and Northeast region; ED-HT: Extreme drought and high temperature in the Brazilian Amazon and Northeast region; ECVI: Extreme Climate Vulnerability Index; SE: Standard Error.

**Fig 3:**
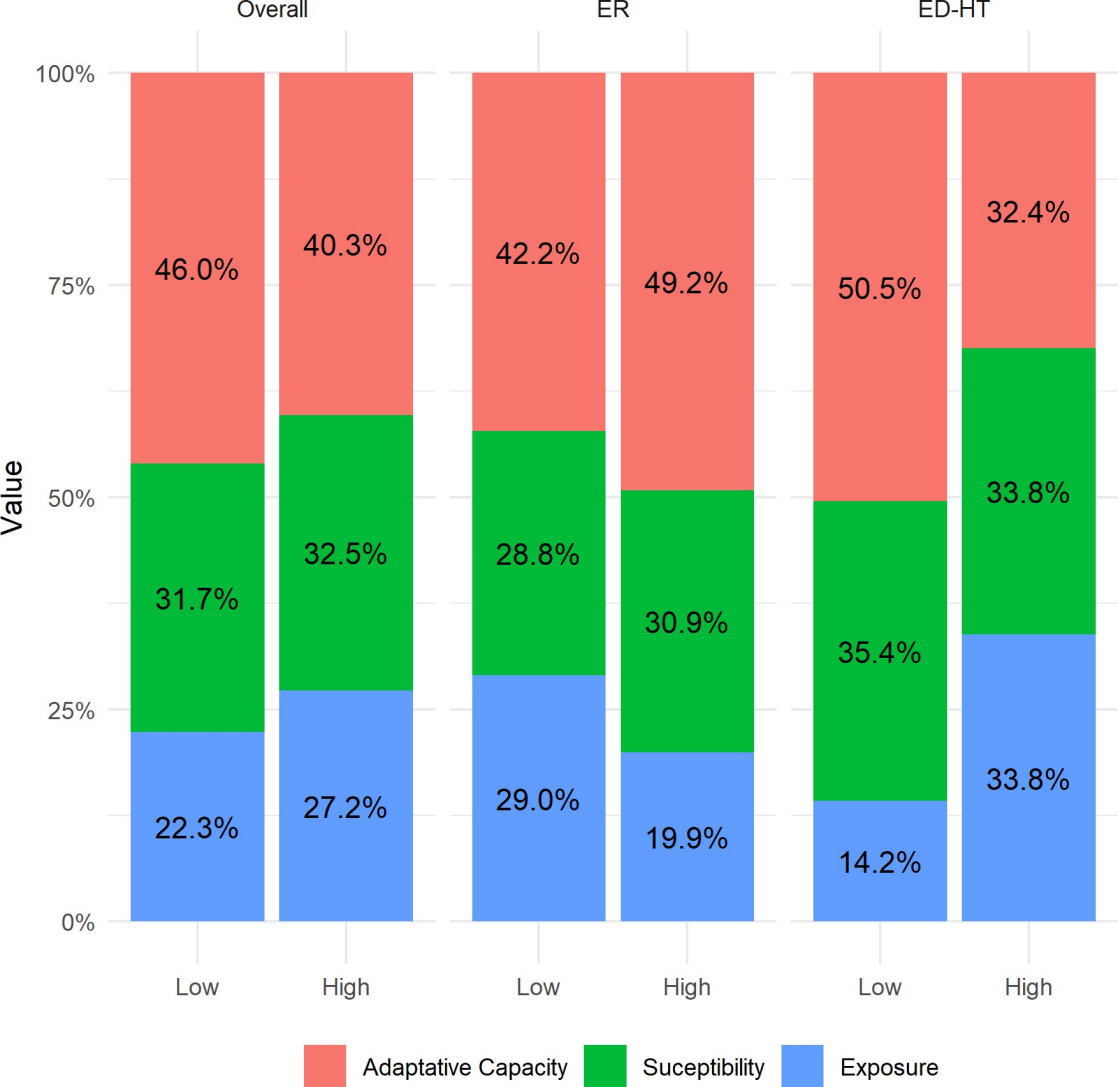
Decomposition analysis of the ECVI for the overall and homoclimatic regions according to the level of climate-sensitive diseases (k = 0.25). ER: Extreme rain zones in the Brazilian Amazon and Northeast region; ED-HT: Extreme drought and high temperature in the Brazilian Amazon and Northeast region; ECVI: Extreme Climate Vulnerability Index.

The ECVI decomposition shows that adaptive capacity and susceptibility are the two most important dimensions, regardless of disease incidence levels (Fig 3). In areas of low disease incidence, adaptive capacity explains 46% of the ECVI, followed by susceptibility (32%). In these areas, the exposure dimension is less important, representing a 22% contribution, compared with a 27% contribution in high disease incidence areas (Fig 3). The relative importance of the exposure dimension in high disease incidence areas is observed among the ED-HT mesoregions (34%), mainly explained by high daily temperature range, extreme wet days, and dry spells. Among the ER mesoregions, adaptive capacity (49%) remains as the main component especially due to the contribution of basic sanitation conditions. These results reflect the nature of infectious and parasitic diseases, which are sensitive to climatic and local sanitary conditions.

## Discussion

Inspired by Alkire and Foster [50], this study proposes a multidimensional index to evaluate health vulnerability to climate extremes (the ECVI) in the Amazonian and Northeastern Brazilian mesoregions. The index includes the three major vulnerability dimensions (exposure, susceptibility, and adaptive capacity), is highly flexible in terms of decomposition and aggregation, and allows for a clear-cut interpretation suitable for policy intervention and surveillance uses. In contrast with previous efforts to measure climate vulnerability, which have used meteorological trends or future climate scenarios [10], our approach utilized extreme climate measures. These are the most appropriate climatic indicators for a vulnerability analysis because climate interacts with health and socioeconomic conditions when extreme events affect the population exposed to these events [2, 59].

To the best of our knowledge, there are only a few studies that have evaluated climatic, demographic, and health conditions for Brazilian regions [2, 51, 59, 79]. Our index differs from previous studies in four main aspects. First, it permits the exact decomposition and disaggregation by dimension and spatial unit, while still providing a simple and direct interpretation. Second, it accounts for the intensity of climatic vulnerability, explicitly estimating separate intensity and prevalence components. Third, it measures uncertainty by including the standard errors for each component. Finally, it incorporates a new classification of regions into homoclimatic zones based on the fuzzy set theory. Our classification uses extreme climate indices for temperature and precipitation, that more appropriately pairs climate with population data.

Measuring the relative importance of climatic variables is particularly important from a policy perspective, as climate extremes have adverse effects on population health and can be mitigated by public policy interventions [2, 59]. The identification of climate vulnerable areas may help improve mitigating actions, such as subsidized credit for areas dependent on non-irrigated agriculture and larger transfers to municipalities in areas lacking climate-robust infrastructure. Furthermore, areas that are close to the vulnerability threshold in our estimates should interpret these findings as a call for preventive action [2]. From the standpoint of the Brazilian Unified Health system, the implementation and expansion of the Family Health Strategy (ESF) has played an essential role in combating diseases typical of tropical climates by monitoring households and providing information to families. Household visits by community health workers are key to detecting possible disease outbreaks and allow for the early identification of infected individuals and for the prompt referral promptly to appropriate health care services [80].

Climatic conditions alone do not explain many disease outbreaks, such as the recent Zika epidemic [81, 82] and the long-established malaria and dengue endemics [83]. Vulnerability to these types of diseases is determined by the interplay between climatic events and structural dimensions, such as existing infrastructure and adequate educational levels [84]. This interaction is particularly relevant in places like Brazil that still suffer from lower levels of overall sanitation coverage and poor socioeconomic conditions, especially in AMZ and the NEB [85].

The use of measurements that map the complex facets of vulnerability in a synthetic index is an appropriate and appealing instrumental choice. Axiomatic multidimensional indices, such as the AF-based ECVI, have additional advantageous features, such as their ease of interpretation and the possibility to make direct comparisons across places and time, as they possess both scale and replication invariance, decomposability, monotonicity, symmetry, normalization, a poverty/deprivation focus, and a weak rearrangement [50]. These axiomatic properties are particularly important to overcome identification issues in multidimensional settings [50, 86]. They are also useful in identifying at-risk areas that should be prioritized by policymakers [87], when explicitly calculating the amount of vulnerability that could be reduced by eliminating one deprivation dimension and in incorporating the degree of vulnerability experienced by those identified as multidimensionally deprived by the dual cutoffs [50].

Although not an exclusive attribute of the ECVI, the underlying methodology supporting our vulnerability measurement allows for the explicit inclusion of two weighting schemes (population size and indicator/dimension weights) and, most importantly, a dual cut-off (one-dimensional and multidimensional). While the one-dimensional cutoff relaxes the nature of the indicators used (qualitative or quantitative), the multidimensional deprivation cutoff correctly imposes simultaneous vulnerability as a multidimensionality and depth criterion for vulnerability experiences. The ECVI weights are exogenously determined [50], differing from vulnerability indices drawn from multivariate techniques, where weights arise from the data’s correlational structure. At first, this may seem like a drawback; however, this is precisely why appropriately chosen weights are key for surveillance purposes over time.

Different methodologies have been used to estimate objective and reliable synthetic measurements of health vulnerability to climate shocks, especially in low and middle-income countries. Although the different measurements proposed are based on varying sets of indicators, they all use the *exposure*, *resilience/adaptive capacity*, and *susceptibility/sensitivity* dimensions to define vulnerability [4]. The commonality of constituent dimensions is not random, but, rather, reflects theoretical efforts to include easy-to-use indicators by decision-makers focused on fighting vulnerability to environmental shocks.

Our ECVI suggests a higher contribution of the exposure dimension in the ED-HT zone in comparison with the ER mesoregions. This result does not come as a surprise, since the ED-HT zone suffers from severe droughts, which subject a large share of its population to vulnerable conditions, especially in semiarid areas [53]. Dry spells affect access to adequate water supplies and create additional barriers for the agricultural sector. These extreme events jeopardize the local population’s socioeconomic wellbeing and food security, since the region is highly dependent on family farming [44].

In the ER homoclimatic zone, adaptive capacity stands out as the most relevant dimension, which reflects the region’s more precarious access to basic sanitation. Noteworthy, this homoclimatic zone comprises 70% of the Brazilian AMZ mesoregions. Our results concur with those found by Menezes et al [2], which analyzed vulnerability for all municipalities in the State of Amazonas. Although these authors applied different methodological procedures and indicators, they found that adaptive capacity and sensitivity provided more prominent contributions than exposure in the study area. Building upon these results, this study shows that this pattern seems to characterize the majority of the Amazonian region, rather than being an isolated feature of the Amazonas State. Besides, these features are very particular to the Brazilian regions that are more exposed to extreme rain.

The analysis of infectious and parasitic disease levels shows a more pronounced vulnerability among regions with high levels of disease incidence. In these areas, the ECVI is around 14% for the entire study area, and there is almost no difference among the ER (13%) and ED-HT (14%) homoclimatic zones. Among low disease incidence mesoregions, these figures are lower than 5%. The decomposition of the ECVI shows that exposure possesses the highest relative importance in explaining vulnerability in areas with a high incidence of infectious and parasitic diseases mainly in the ED-HT homoclimatic zone. The adverse health effects of climatic extremes are well documented in the empirical literature [1, 2, 59, 22, 23]. Furthermore, adverse climatic conditions can contribute to an increase in climate-sensitive diseases and intensify symptoms of several other diseases, especially among the population groups most susceptible to extreme climate events, such as the elderly and children [22, 77].

For the ER homoclimatic zone, adaptive capacity (49%) followed by susceptibility (31%) were the most important components to explain multidimensional vulnerability among mesoregions with high incidence of infectious and parasitic diseases. Even though exposure to extreme climatic events only accounts for 20% of the overall vulnerability, the interplay between climate and poor basic sanitary conditions can exacerbate health vulnerability. In places where infrastructure or adaptive measures are inadequate, these impacts can be more severe, ultimately leading to loss of human life [2, 4, 22, 59]. Thus, identifying socioenvironmental deprivation and exposure to these events is key for decision makers to properly address a population’s health vulnerabilities and establish strategies to mitigate adverse effects on climate-sensitive diseases [88].

Despite enhancing the literature on health vulnerability to climate extremes, our findings have some limitations. Using hospitalization rates as a proxy for infectious and parasitic disease incidence is a somewhat restrictive health measurement, as it mainly reflects more severe cases of infectious disease. Epidemiological profiles at the municipal level can be constructed using four different data sources in Brazil, including hospitalizations (SIH/AIH), mortality (SIM), disease notification (SINAN), and ambulatory care (SIA). Providing patient level data to the SIA is not mandatory, which impedes the development of accurate epidemiological profiles [62]. While SINAN data recording is mandatory, its coverage is still precarious, especially for the Brazilian Amazon and Northeast region [89]. Although mortality data is more commonly recorded, their use is even more restrictive than hospitalization data. In general, hospitalization information is viewed as having elevated coverage in Brazil because payments for inpatient procedures are contingent upon recording them in the SIH system. Furthermore, epidemiological data at the patient level is available through the SIH, making it the most used information source, along with mortality, for studies on population health vulnerability [90].

A second limitation regards spatial resolution, since we use mesoregions as our units of analysis. We believe that this aggregation level is not an important caveat as intra-mesoregion heterogeneities are less striking in our study areas when compared to other Brazilian regions, such as the Southeast [37, 45, 53]. Our results also place limited emphasis on how climatic conditions can affect population health because of the cross-sectional and correlational nature of our approach. However, our empirical strategy still provides useful insights into regional disparities in population vulnerabilities, facilitating the prioritization of regions experiencing greater health sensitivity to poor sociodemographic conditions and extreme climate events. The dimensional decomposition of the ECVI is particularly helpful in this regard, as it allows for the identification of the areas that most contribute to explain each study region’s vulnerability and how much this vulnerability can be reduced by eliminating specific deprivation factors in those areas.

In this study we use climate data developed by Xavier et al [57], which provides greater meteorological information accuracy and coverage than data from the National Institute of Meteorology (INMET). Moreover, the use of a daily time-based series that encompasses at least 30 years of information is more suitable to measure climate extremes than the use of short term or higher temporal resolution precipitation and temperature series, such as monthly or yearly measurements.

The use of appropriate climatic markers when studying population vulnerability is even more relevant in areas frequently affected by climatic extremes [53, 91]. The increase in the frequency and magnitude of climate extremes in Brazilian Amazonia and in the Northeast highlights the importance of combining information on hydrometeorological and climatological phenomena. The mere use of temperature and precipitation levels does not fully reflect the intensity and the frequency of climatic events in both regions, both of which will likely intensify in the future [92, 93].

Despite the proliferation of indices and methodological approaches to measuring vulnerability in the literature [4], our AF-based vulnerability index is easy to use, generalizable to other areas, and well-suited for monitoring vulnerability over time. Other indices inspired by the Human Development Index, such as the one proposed by Silveira Marinho et al [53], are not decomposable and lack other axiomatic features that are highly desirable for multidimensional indices, which is particularly true for the monotonicity and focus axioms in a multidimensional setting. Furthermore, the ECVI’s added complexity does not come at a cost of greater difficulties to obtain and/or interpret the index. The AF method is a solid approach and is widely recognized as being instrumental for public policymaking and being easy to interpret. In the future, we plan to enhance the identification of the multidimensional cutoff with non-parametric solutions, increasing the robustness of this method even further. Finally, future analyses will also incorporate downscaled climate data that encompass all Brazilian regions.

## Supporting information

S1 Fig Radar charts of average ECVI indicators values by homoclimatic zone. TXx: Monthly maximum value of daily maximum temperature (oC), TNx: Monthly maximum value of daily minimum temperature (oC); TX90p: Percentage of warm days; TN90p: Percentage of warm nights; DTR: Daily temperature range; Cdd: Dry spell; R99p: Extremely wet days; ER: Extreme rain zones in the Brazilian Amazon and Northeast region; ED-HT: Extreme drought and high temperature in the Brazilian Amazon and Northeast region; ECVI: Extreme Climate Vulnerability Index.
(TIF)

S2 Fig Sensitivity and dominance analyses to define the cut-off point (k) for the ECVI index. ER: Extreme rain zones in the Brazilian Amazon and Northeast region; ED-HT: Extreme drought and high temperature in the Brazilian Amazon and Northeast region; ECVI: Extreme Climate Vulnerability Index.
(TIF)

S3 Fig Boxplot of the posterior distributions of *λ*_*kjl*_ for each extreme climate index included in the GoM model. TXx: Monthly maximum value of daily maximum temperature (oC), TNx: Monthly maximum value of daily minimum temperature (oC); TX90p: Percentage of warm days; TN90p: Percentage of warm nights; DTR: Daily temperature range; Cdd: Dry spell; R99p: Extremely wet days; Profile 1—ER: Extreme rain zones in the Brazilian Amazon and Northeast region; Profile 2—ED-HT: Extreme drought and high temperature in the Brazilian Amazon and Northeast region; ECVI: Extreme Climate Vulnerability Index; Q1-Q5: Quantiles of the extreme climate index distribution.
(TIF)

S1 Table Descriptive statistics and vulnerability cutoff-points of ECVI indicators by the homoclimatic zone. ER: Extreme rain zones in the Brazilian Amazon and Northeast region; ED-HT: Extreme drought and high temperature in the Brazilian Amazon and Northeast region; ECVI: Extreme Climate Vulnerability Index. (1) ER: 10.6 –ED-HT: 11.6. (2) ER: 10.4 –ED-HT: 9.3. (3) Brazilian Currency was converted to the 2010 US dollars exchange rate using the CCEMG—EPPI-Center Cost Converter website (<http://eppi.ioe.ac.uk/costconversion/default.aspx>). (4) Low incidence areas: 1^st^ quantile of the rate of infectious diseases distribution; High incidence areas: 4^th^ and 5^th^ quantiles of the rate of infectious diseases distribution.
(DOCX)

S2 Table **1**. Sensitivity analysis to define the cut-off point (k) for the ECVI index, Overall study area. ECVI: Extreme Climate Vulnerability Index. **2.** Sensitivity analysis to define the cut-off point (k) for the ECVI index, ER homoclimatic zone. ER: Extreme rain zones in the Brazilian Amazon and Northeast region; ECVI: Extreme Climate Vulnerability Index. **3.** Sensitivity analysis to define the cut-off point (k) for the ECVI index, ED-HT homoclimatic zone. ED-HT: Extreme drought and high temperature in the Brazilian Amazon and Northeast region; ECVI: Extreme Climate Vulnerability Index.
(DOCX)

S3 Table **1.** ECVI, Censored Headcount, and Vulnerability Intensity for the Overall and homoclimatic zones (k = 0.30). ER: Extreme rain zones in the Brazilian Amazon and Northeast region; ED-HT: Extreme drought and high temperature in the Brazilian Amazon and Northeast region; ECVI: Extreme Climate Vulnerability Index. **2.** Decomposition analysis of the ECVI for the homoclimatic zone in the Brazilian Amazon and the Northeast (k = 0.30). ER: Extreme rain zones in the Brazilian Amazon and Northeast region; ED-HT: Extreme drought and high temperature in the Brazilian Amazon and Northeast region; ECVI: Extreme Climate Vulnerability Index.
(DOCX)
