# Supplementary figures and images for: Health-related vulnerability to climate extremes in homoclimatic zones of Amazonia and Northeast region of Brazil
Source: PLoS One. 2021 Nov 11;16(11):e0259780. doi: 10.1371/journal.pone.0259780 (PMC8584767; doi:10.1371/journal.pone.0259780)

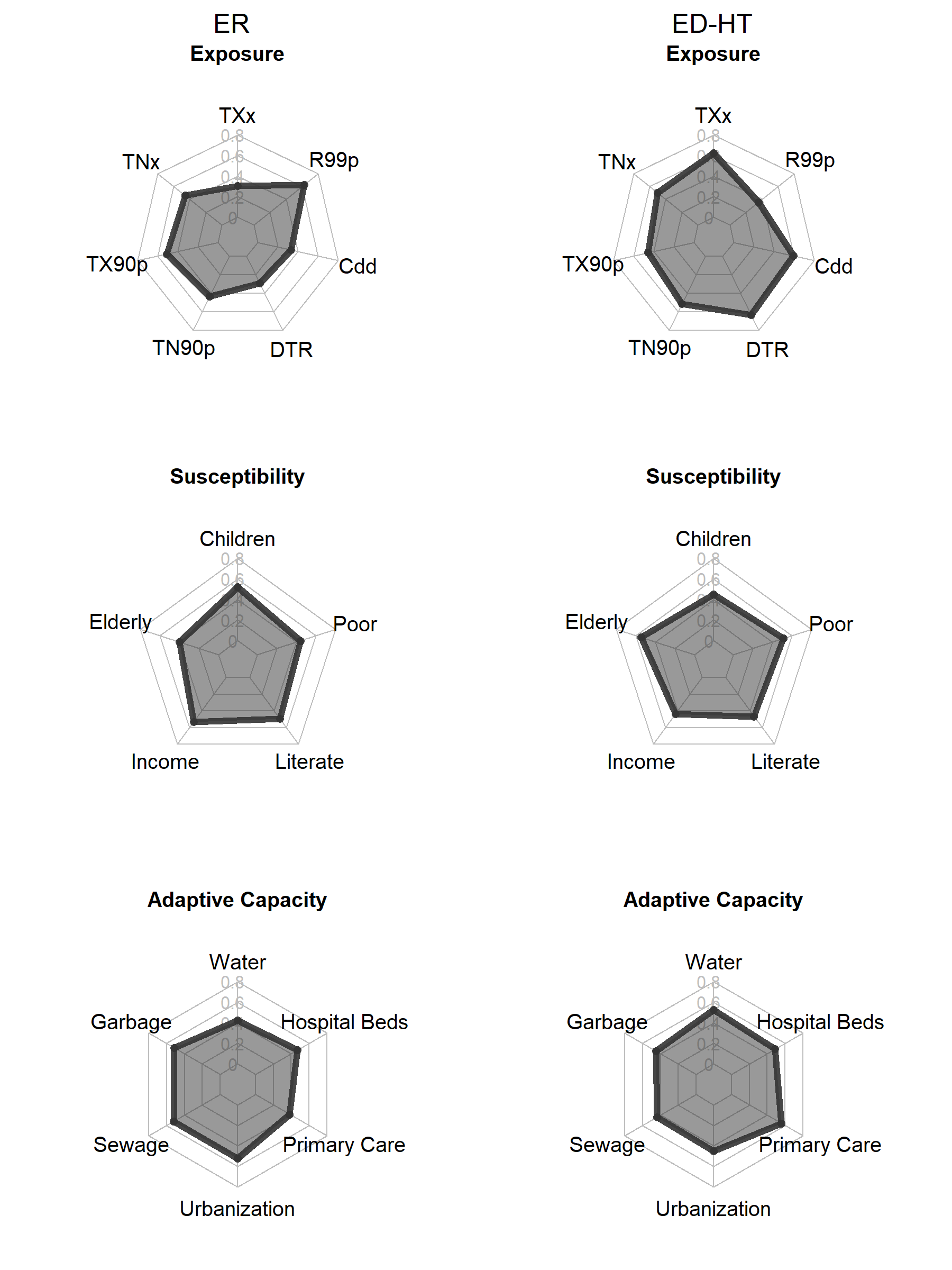

Supplement: S1 Fig — TXx: Monthly maximum value of daily maximum temperature (oC), TNx: Monthly maximum value of daily minimum temperature (oC); TX90p: Percentage of warm days; TN90p: Percentage of warm nights; DTR: Daily temperature range; Cdd: Dry spell; R99p: Extremely wet days; ER: Extreme rain zones in the Brazilian Amazon and Northeast region; ED-HT: Extreme drought and high temperature in the Brazilian Amazon and Northeast region; ECVI: Extreme Climate Vulnerability Index. (TIF) [file pone.0259780.s001.tif]

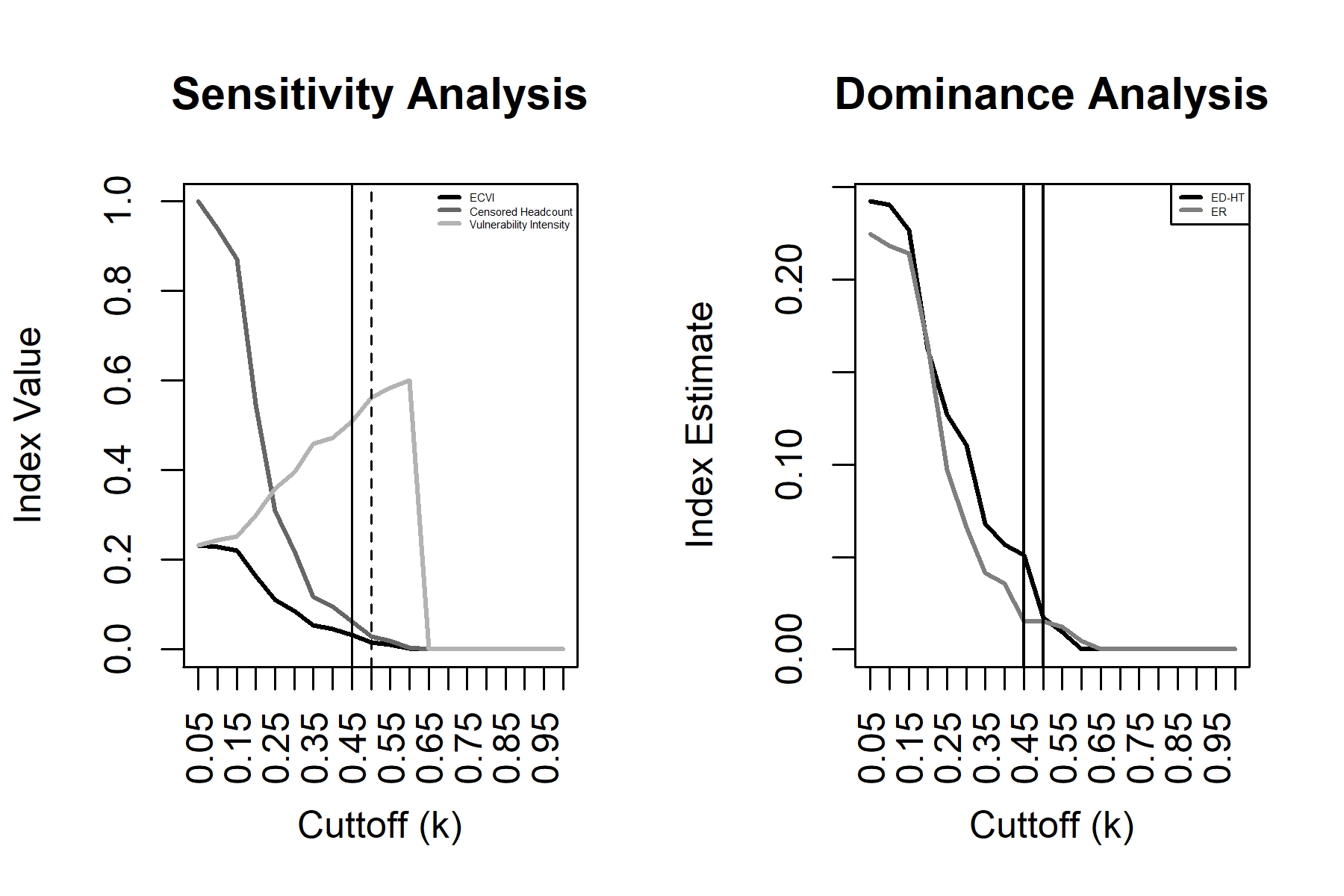

Supplement: S2 Fig — ER: Extreme rain zones in the Brazilian Amazon and Northeast region; ED-HT: Extreme drought and high temperature in the Brazilian Amazon and Northeast region; ECVI: Extreme Climate Vulnerability Index. (TIF) [file pone.0259780.s002.tif]

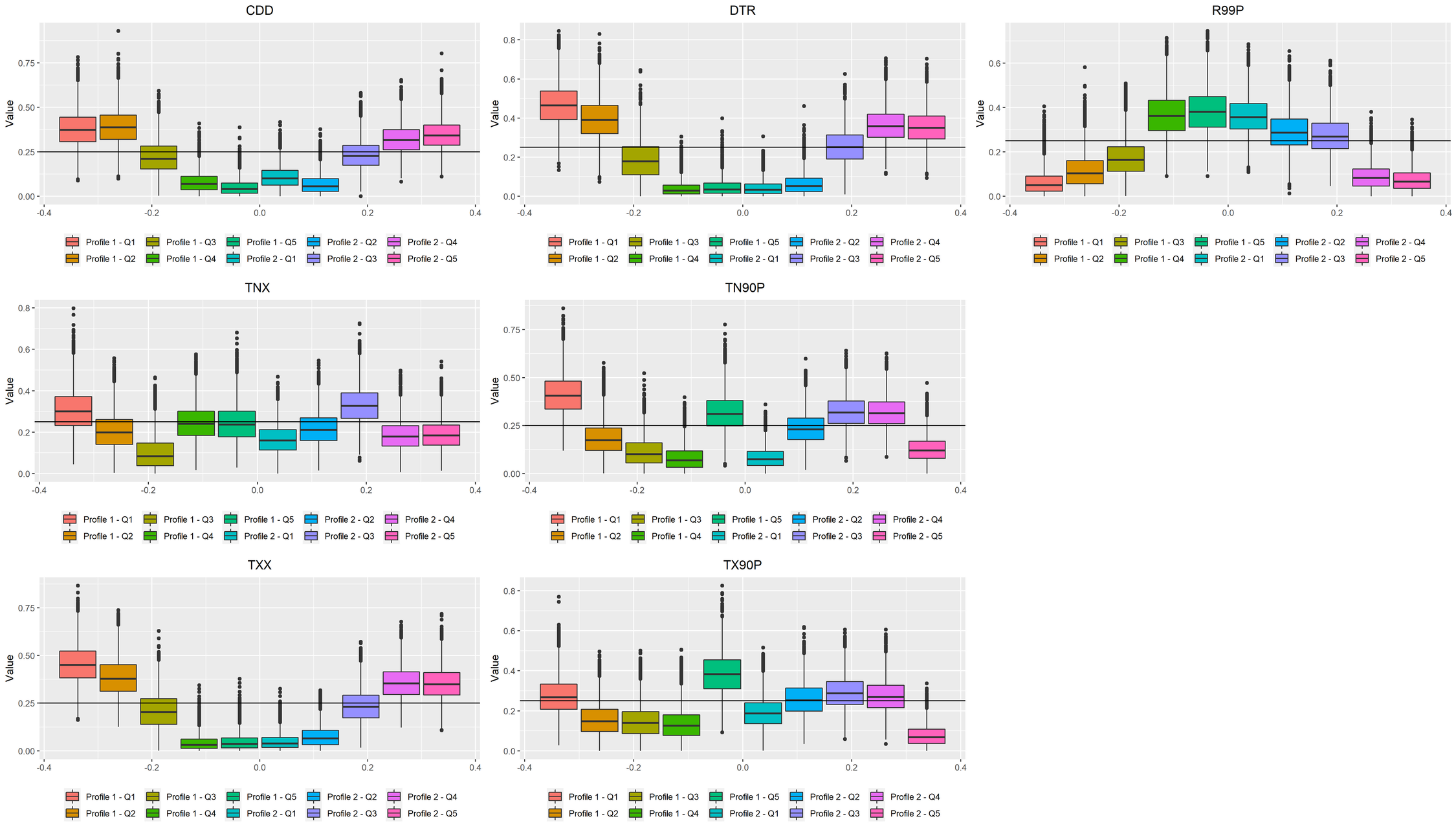

Supplement: S3 Fig — TXx: Monthly maximum value of daily maximum temperature (oC), TNx: Monthly maximum value of daily minimum temperature (oC); TX90p: Percentage of warm days; TN90p: Percentage of warm nights; DTR: Daily temperature range; Cdd: Dry spell; R99p: Extremely wet days; Profile 1—ER: Extreme rain zones in the Brazilian Amazon and Northeast region; Profile 2—ED-HT: Extreme drought and high temperature in the Brazilian Amazon and Northeast region; ECVI: Extreme Climate Vulnerability Index; Q1-Q5: Quantiles of the extreme climate index distribution. (TIF) [file pone.0259780.s003.tif]
