# Supplementary material for: Health-related vulnerability to climate extremes in homoclimatic zones of Amazonia and Northeast region of Brazil
Source: PLoS One. 2021 Nov 11;16(11):e0259780. doi: 10.1371/journal.pone.0259780 (PMC8584767; doi:10.1371/journal.pone.0259780)
Supplement: S1 Table — ER: Extreme rain zones in the Brazilian Amazon and Northeast region; ED-HT: Extreme drought and high temperature in the Brazilian Amazon and Northeast region; ECVI: Extreme Climate Vulnerability Index. (1) ER: 10.6 –ED-HT: 11.6. (2) ER: 10.4 –ED-HT: 9.3. (3) Brazilian Currency was converted to the 2010 US dollars exchange rate using the CCEMG—EPPI-Center Cost Converter website (<http://eppi.ioe.ac.uk/costconversion/default.aspx>). (4) Low incidence areas: 1st quantile of the rate of infectious diseases distribution; High incidence areas: 4th and 5th quantiles of the rate of infectious diseases distribution. (DOCX) [file pone.0259780.s004.docx]

**S1 Table. Descriptive Statistics and vulnerability cutoff-points of the indicators that compose the ECVI for the homoclimatic region**

|  | **ER** | | | **ED-HT** | | | **Avg. Dif.** | **p-value** | **Cutoff-point** |
| --- | --- | --- | --- | --- | --- | --- | --- | --- | --- |
| **Indicator** | **Mean** | **Min** | **Max** | **Mean** | **Min** | **Max** |  |  |  |
| **Exposure** |  |  |  |  |  |  |  |  |  |
| Monthly maximum value of daily maximum temperature (^o^C) | 35.3 | 32.0 | 39.2 | 37.8 | 32.4 | 39.8 | -2.5 | 0.0000 | 37.7 |
| Monthly maximum value of daily minimum temperature (^o^C) | 24.8 | 21.3 | 27.0 | 25.1 | 22.3 | 26.4 | -0.2 | 0.4283 | 25.8 |
| Percentage of warm days | 18.2 | 0.4 | 52.7 | 15.3 | 2.9 | 26.9 | 2.9 | 0.2188 | 22.4 |
| Percentage of warm nights | 16.8 | 0.4 | 44.9 | 16.8 | 4.7 | 31.5 | 0.1 | 0.9843 | 21.6 |
| Daily temperature range | 8.8 | 6.7 | 12.2 | 10.9 | 7.3 | 13.7 | -2.1 | 0.0000 | 10.9 |
| Dry spell | 30.0 | 6.7 | 101.3 | 69.5 | 4.3 | 155.3 | -39.5 | 0.0000 | 72.3 |
| Extremely wet days | 170.3 | 50.2 | 337.0 | 76.5 | 0.0 | 202.0 | 93.9 | 0.0000 | 170.3 |
| **Susceptibility** |  |  |  |  |  |  |  |  |  |
| Proportion of elderly (%) | 9.0 | 4.7 | 14.2 | 10.0 | 5.1 | 13.8 | -1.0 | 0.0540 | 11.0 ^(1)^ |
| Proportion of children (%) | 8.5 | 6.8 | 13.0 | 8.3 | 6.7 | 14.2 | 0.2 | 0.5222 | 10.0 ^(2)^ |
| Household per capita income (R$ (US$^(3)^)) | 500.3  (361.0) | 201.8  (145.6) | 753.6  (543.7) | 429.1  (309.6) | 206.7  (149.1) | 769.2  (555.0) | 71.2 | 0.1167 | 296.8  (214.1) |
| Proportion of Poor individuals | 53.0 | 31.4 | 78.0 | 57.3 | 37.5 | 79.3 | -4.4 | 0.1239 | 66.4 |
| Proportion of literate adults (%) | 42.6 | 24.4 | 53.3 | 39.7 | 23.4 | 54.8 | 2.9 | 0.1184 | 34.5 |
| **Adaptive Capacity** |  |  |  |  |  |  |  |  |  |
| Proportion of household with adequate sewage (%) | 44.5 | 10.9 | 67.1 | 38.4 | 11.6 | 80.3 | 6.0 | 0.2077 | 18.3 |
| Proportion of household with adequate water supply (%) | 69.1 | 18.5 | 92.4 | 73.4 | 41.9 | 93.8 | -4.3 | 0.2664 | 57.2 |
| Proportion of household with garbage collection (%) | 77.8 | 42.0 | 95.4 | 69.9 | 43.8 | 92.0 | 7.9 | 0.0401 | 58.1 |
| Urbanization rate (%) | 76.8 | 43.4 | 97.3 | 69.1 | 47.3 | 93.5 | 7.8 | 0.0447 | 57.8 |
| Primary care coverage | 81.2 | 51.5 | 103.9 | 90.6 | 52.9 | 119.9 | -9.4 | 0.0388 | 80.2 |
| Hospital beds per 100,000 inhabitants | 222.3 | 82.1 | 317.3 | 216.9 | 93.5 | 291.0 | 5.4 | 0.6794 | 162.2 |
| Rate of infectious diseases (per 100,000) | 707.9 | 313.1 | 1584.4 | 814.5 | 198.7 | 1705.5 | -106.6 | 0.2230 | Low incidence ^(4)^:  ≤470.4  High incidence^(4)^: >933.0 |

ER: Extreme rain zones in the Brazilian Amazon and Northeast region; ED-HT: Extreme drought and high temperature in the Brazilian Amazon and Northeast region; ECVI: Extreme Climate Vulnerability Index.

1. ER: 10.6 – ED-HT: 11.6
2. ER: 10.4 – ED-HT: 9.3
3. Brazilian Currency was converted to the 2010 US dollars exchange rate using the CCEMG - EPPI-Center Cost Converter website (<http://eppi.ioe.ac.uk/costconversion/default.aspx>).
4. Low incidence areas: 1^st^ quantile of the rate of infectious diseases distribution; High incidence areas: 4^th^ and 5^th^ quantiles of the rate of infectious diseases distribution
