# Supplementary material for: Health-related vulnerability to climate extremes in homoclimatic zones of Amazonia and Northeast region of Brazil
Source: PLoS One. 2021 Nov 11;16(11):e0259780. doi: 10.1371/journal.pone.0259780 (PMC8584767; doi:10.1371/journal.pone.0259780)
Supplement: S2 Table — 1. Sensitivity analysis to define the cut-off point (k) for the ECVI index, Overall study area. ECVI: Extreme Climate Vulnerability Index. 2. Sensitivity analysis to define the cut-off point (k) for the ECVI index, ER homoclimatic zone. ER: Extreme rain zones in the Brazilian Amazon and Northeast region; ECVI: Extreme Climate Vulnerability Index. 3. Sensitivity analysis to define the cut-off point (k) for the ECVI index, ED-HT homoclimatic zone. ED-HT: Extreme drought and high temperature in the Brazilian Amazon and Northeast region; ECVI: Extreme Climate Vulnerability Index. (DOCX) [file pone.0259780.s005.docx]

**S2.1 Table. Sensitivity Analysis to define the cut-off point (k) for the ECVI index, Overall study regions**

| **K** | **ECVI** | | **Censored Headcount** | | **Vulnerability Intensity** | |
| --- | --- | --- | --- | --- | --- | --- |
|  | **Index** | **SE** | **Index** | **SE** | **Index** | **SE** |
| 0.05 | 0.233 | 0.014 | 1.000 | 0.000 | 0.233 | 0.014 |
| 0.10 | 0.229 | 0.015 | 0.939 | 0.037 | 0.244 | 0.014 |
| 0.15 | 0.220 | 0.017 | 0.871 | 0.054 | 0.253 | 0.014 |
| 0.20 | 0.164 | 0.024 | 0.547 | 0.083 | 0.299 | 0.017 |
| **0.25** | **0.111** | **0.024** | **0.309** | **0.070** | **0.359** | **0.022** |
| 0.30 | 0.087 | 0.021 | 0.218 | 0.053 | 0.396 | 0.017 |
| 0.35 | 0.054 | 0.018 | 0.117 | 0.041 | 0.459 | 0.020 |
| 0.40 | 0.045 | 0.017 | 0.096 | 0.038 | 0.473 | 0.024 |
| 0.45 | 0.032 | 0.014 | 0.063 | 0.028 | 0.509 | 0.023 |
| 0.50 | 0.016 | 0.008 | 0.029 | 0.013 | 0.562 | 0.013 |
| 0.55 | 0.011 | 0.007 | 0.019 | 0.011 | 0.584 | 0.006 |
| 0.60 | 0.002 | 0.003 | 0.004 | 0.004 | 0.602 | 0.000 |
| 0.65 | 0.000 | 0.000 | 0.000 | 0.000 | 0.000 | 0.000 |
| 0.70 | 0.000 | 0.000 | 0.000 | 0.000 | 0.000 | 0.000 |
| 0.75 | 0.000 | 0.000 | 0.000 | 0.000 | 0.000 | 0.000 |
| 0.80 | 0.000 | 0.000 | 0.000 | 0.000 | 0.000 | 0.000 |
| 0.85 | 0.000 | 0.000 | 0.000 | 0.000 | 0.000 | 0.000 |
| 0.90 | 0.000 | 0.000 | 0.000 | 0.000 | 0.000 | 0.000 |
| 0.95 | 0.000 | 0.000 | 0.000 | 0.000 | 0.000 | 0.000 |
| 1.00 | 0.000 | 0.000 | 0.000 | 0.000 | 0.000 | 0.000 |

ECVI: Extreme Climate Vulnerability Index.

**S2.2 Table. Sensitivity Analysis to define the cut-off point (k) for the ECVI index, ER homoclimatic region**

| **K** | **ECVI** | | **Censored Headcount** | | **Vulnerability Intensity** | |
| --- | --- | --- | --- | --- | --- | --- |
|  | **Index** | **SE** | **Index** | **SE** | **Index** | **SE** |
| 0.05 | 0.225 | 0.018 | 1.000 | 0.000 | 0.225 | 0.018 |
| 0.10 | 0.218 | 0.021 | 0.908 | 0.065 | 0.241 | 0.016 |
| 0.15 | 0.214 | 0.022 | 0.872 | 0.073 | 0.246 | 0.016 |
| 0.20 | 0.165 | 0.032 | 0.595 | 0.118 | 0.277 | 0.021 |
| **0.25** | **0.097** | **0.033** | **0.284** | **0.106** | **0.342** | **0.033** |
| 0.30 | 0.066 | 0.026 | 0.167 | 0.066 | 0.394 | 0.023 |
| 0.35 | 0.041 | 0.023 | 0.092 | 0.054 | 0.450 | 0.035 |
| 0.40 | 0.036 | 0.022 | 0.077 | 0.051 | 0.460 | 0.044 |
| 0.45 | 0.015 | 0.010 | 0.026 | 0.017 | 0.567 | 0.017 |
| 0.50 | 0.015 | 0.010 | 0.026 | 0.017 | 0.567 | 0.017 |
| 0.55 | 0.012 | 0.009 | 0.021 | 0.016 | 0.584 | 0.010 |
| 0.60 | 0.005 | 0.005 | 0.008 | 0.008 | 0.602 | 0.000 |
| 0.65 | 0.000 | 0.000 | 0.000 | 0.000 | 0.000 | 0.000 |
| 0.70 | 0.000 | 0.000 | 0.000 | 0.000 | 0.000 | 0.000 |
| 0.75 | 0.000 | 0.000 | 0.000 | 0.000 | 0.000 | 0.000 |
| 0.80 | 0.000 | 0.000 | 0.000 | 0.000 | 0.000 | 0.000 |
| 0.85 | 0.000 | 0.000 | 0.000 | 0.000 | 0.000 | 0.000 |
| 0.90 | 0.000 | 0.000 | 0.000 | 0.000 | 0.000 | 0.000 |
| 0.95 | 0.000 | 0.000 | 0.000 | 0.000 | 0.000 | 0.000 |
| 1.00 | 0.000 | 0.000 | 0.000 | 0.000 | 0.000 | 0.000 |

ER: Extreme rain zones in the Brazilian Amazon and Northeast region; ECVI: Extreme Climate Vulnerability Index.

**S2.3 Table. Sensitivity Analysis to define the cut-off point (k) for the ECVI index, ED-HT homoclimatic region**

| **K** | **ECVI** | | **Censored Headcount** | | **Vulnerability Intensity** | |
| --- | --- | --- | --- | --- | --- | --- |
|  | **Index** | **SE** | **Index** | **SE** | **Index** | **SE** |
| 0.05 | 0.242 | 0.022 | 1.000 | 0.000 | 0.242 | 0.022 |
| 0.10 | 0.241 | 0.022 | 0.974 | 0.026 | 0.247 | 0.023 |
| 0.15 | 0.227 | 0.027 | 0.869 | 0.081 | 0.261 | 0.024 |
| 0.20 | 0.163 | 0.038 | 0.492 | 0.111 | 0.330 | 0.024 |
| **0.25** | **0.127** | **0.036** | **0.337** | **0.093** | **0.376** | **0.023** |
| 0.30 | 0.110 | 0.034 | 0.278 | 0.085 | 0.398 | 0.024 |
| 0.35 | 0.068 | 0.029 | 0.146 | 0.064 | 0.465 | 0.021 |
| 0.40 | 0.057 | 0.027 | 0.118 | 0.057 | 0.482 | 0.020 |
| 0.45 | 0.051 | 0.027 | 0.104 | 0.056 | 0.492 | 0.022 |
| 0.50 | 0.017 | 0.012 | 0.031 | 0.021 | 0.557 | 0.019 |
| 0.55 | 0.009 | 0.010 | 0.016 | 0.017 | 0.586 | 0.000 |
| 0.60 | 0.000 | 0.000 | 0.000 | 0.000 | 0.000 | 0.000 |
| 0.65 | 0.000 | 0.000 | 0.000 | 0.000 | 0.000 | 0.000 |
| 0.70 | 0.000 | 0.000 | 0.000 | 0.000 | 0.000 | 0.000 |
| 0.75 | 0.000 | 0.000 | 0.000 | 0.000 | 0.000 | 0.000 |
| 0.80 | 0.000 | 0.000 | 0.000 | 0.000 | 0.000 | 0.000 |
| 0.85 | 0.000 | 0.000 | 0.000 | 0.000 | 0.000 | 0.000 |
| 0.90 | 0.000 | 0.000 | 0.000 | 0.000 | 0.000 | 0.000 |
| 0.95 | 0.000 | 0.000 | 0.000 | 0.000 | 0.000 | 0.000 |
| 1.00 | 0.000 | 0.000 | 0.000 | 0.000 | 0.000 | 0.000 |

ED-HT: Extreme drought and high temperature in the Brazilian Amazon and Northeast region; ECVI: Extreme Climate Vulnerability Index.
