# Supplementary material for: Health-related vulnerability to climate extremes in homoclimatic zones of Amazonia and Northeast region of Brazil
Source: PLoS One. 2021 Nov 11;16(11):e0259780. doi: 10.1371/journal.pone.0259780 (PMC8584767; doi:10.1371/journal.pone.0259780)
Supplement: S3 Table — 1. ECVI, Censored Headcount, and Vulnerability Intensity for the Overall and homoclimatic zones (k = 0.30). ER: Extreme rain zones in the Brazilian Amazon and Northeast region; ED-HT: Extreme drought and high temperature in the Brazilian Amazon and Northeast region; ECVI: Extreme Climate Vulnerability Index. 2. Decomposition analysis of the ECVI for the homoclimatic zone in the Brazilian Amazon and the Northeast (k = 0.30). ER: Extreme rain zones in the Brazilian Amazon and Northeast region; ED-HT: Extreme drought and high temperature in the Brazilian Amazon and Northeast region; ECVI: Extreme Climate Vulnerability Index. (DOCX) [file pone.0259780.s006.docx]

**S3.1 Table. ECVI, Censored Headcount and Vulnerability Intensity for the Overall and homogeclimatic regions (k=0.30)**

| **Indicator** | **Overall** | | **ER** | | **ED-HT** | |
| --- | --- | --- | --- | --- | --- | --- |
|  | **Index** | **SE** | **Index** | **SE** | **Index** | **SE** |
| ECVI | 0.087 | 0.022 | 0.066 | 0.026 | 0.110 | 0.034 |
| Headcount (H) | 0.218 | 0.053 | 0.167 | 0.066 | 0.278 | 0.085 |
| Intensity (A) | 0.396 | 0.017 | 0.394 | 0.023 | 0.398 | 0.024 |

ER: Extreme rain zones in the Brazilian Amazon and Northeast region; ED-HT: Extreme drought and high temperature in the Brazilian Amazon and Northeast region; ECVI: Extreme Climate Vulnerability Index.

**S3.2 Table. Decomposition analysis of the ECVI for the homoclimatic regions in the Brazilian Amazon and Northeast (k=0.30)**

| **Dimension/Indicator** | **Overall** | | **ER** | | **ED-HT** | |
| --- | --- | --- | --- | --- | --- | --- |
|  | **Relative Contribution** | **SE** | **Relative Contribution** | **SE** | **Relative Contribution** | **SE** |
| **Exposure** | **32.7** |  | **24.3** |  | **38.6** |  |
| Monthly maximum value of daily maximum temperature (^o^C) | 5.0 | 0.015 | 2.8 | 0.016 | 6.5 | 0.020 |
| Monthly maximum value of daily minimum temperature (^o^C) | 4.1 | 0.014 | 1.5 | 0.014 | 5.9 | 0.019 |
| Percentage of warm days | 4.1 | 0.013 | 4.3 | 0.021 | 4.0 | 0.017 |
| Percentage of warm nights | 3.8 | 0.013 | 4.3 | 0.021 | 3.4 | 0.016 |
| Daily temperature range | 6.8 | 0.014 | 6.4 | 0.023 | 7.0 | 0.018 |
| Dry spell | 4.1 | 0.014 | 1.8 | 0.013 | 5.7 | 0.019 |
| Extremely wet days | 4.8 | 0.015 | 3.2 | 0.017 | 6.0 | 0.020 |
| **Susceptibility** | **30.1** |  | **28.2** |  | **31.4** |  |
| Higher proportion of elderly | 1.9 | 0.012 | 1.2 | 0.013 | 2.3 | 0.018 |
| Higher proportion of children | 7.9 | 0.019 | 10.7 | 0.027 | 5.9 | 0.024 |
| Average per capita income below 1/2 minimum wage | 6.2 | 0.020 | 6.2 | 0.034 | 6.1 | 0.024 |
| Higher proportion of poor individuals | 7.3 | 0.021 | 7.3 | 0.033 | 7.3 | 0.028 |
| Lower proportion of literate adults | 6.9 | 0.019 | 2.7 | 0.016 | 9.7 | 0.024 |
| **Adaptive Capacity** | **37.2** |  | **47.5** |  | **30.0** |  |
| Lower proportion of households with adequate sewage | 7.1 | 0.016 | 7.4 | 0.024 | 6.9 | 0.020 |
| Lower proportion of households with adequate water supply | 6.4 | 0.017 | 12.2 | 0.014 | 2.3 | 0.015 |
| Lower proportion of households with garbage collection | 9.0 | 0.013 | 9.3 | 0.021 | 8.8 | 0.017 |
| Lower levels of urbanization | 7.7 | 0.016 | 8.6 | 0.022 | 7.0 | 0.021 |
| Lower coverage of primary care | 2.6 | 0.011 | 5.4 | 0.025 | 0.7 | 0.006 |
| Lower rates of hospital bed per 100,000 | 4.3 | 0.014 | 4.5 | 0.021 | 4.2 | 0.019 |

ER: Extreme rain zones in the Brazilian Amazon and Northeast region; ED-HT: Extreme drought and high temperature in the Brazilian Amazon and Northeast region; ECVI: Extreme Climate Vulnerability Index.
